# The Impact of Shift-Work and Night Shift-Work on Thyroid: A Systematic Review

**DOI:** 10.3390/ijerph17051527

**Published:** 2020-02-27

**Authors:** Veruscka Leso, Ilaria Vetrani, Alessandra Sicignano, Rosaria Romano, Ivo Iavicoli

**Affiliations:** Section of Occupational Medicine, Department of Public Health, University of Naples Federico II, Via S. Pansini 5, 80131 Naples, Italy; veruscka.leso@unina.it (V.L.); ilariavetrani20@gmail.com (I.V.); alessandra.sicignano@yahoo.it (A.S.); rosaria_romano@libero.it (R.R.)

**Keywords:** shift-work, night-work, work schedule, thyroid, thyroid hormones, occupational risk factors, risk assessment, risk management, 24-h economy, aging

## Abstract

Thyroid hormones are regulated by the pituitary thyroid stimulating hormone (TSH), whose secretion presents a circadian rhythmicity. Indeed, it is conceivable that shift- and night shift-work, affecting sleep-wake rhythms, may impact thyroid functionality. Therefore, the aim of the present review was to provide an overview on the association between shift- and night shift-work and thyroid hormonal changes and disease development. A systematic review of studies available in PubMed, Scopus, and ISI Web of Science databases was performed. A positive association between night shift-work and increased TSH concentrations was reported by most of the reviewed investigations. Inconclusive evidence was available on thyroid diseases. However, the limited number of studies, the noticeable heterogeneity in the shift-work scheduling, in terms of amount, duration, type of shift- or night shift-work, prevents easily integrating findings and extrapolating definite conclusions. Further investigation seems necessary to better define the relationship between shift schedules and different thyroid outcomes, and possible long-term implications of early functional changes. Overall, this may support the adoption of advanced risk assessment and management strategies aimed to achieve a safer workplace organization and a timely, responsible realization of all the benefits of a 24-h economy.

## 1. Introduction

The thyroid gland produces hormones, triiodothyronine (T3) and thyroxine (T4), that are essential for body’s metabolism, heat production, suitable development and differentiation of cells, and growth [1]. The hypothalamic-pituitary-thyroid (HPT) axis controls the thyroid function, as the T3 and T4 hormones are secreted in response to the pituitary thyroid stimulating hormone (TSH) [2]. Thyroid stimulating hormone secretion exhibits a clear daily rhythmicity, related to the sleep-wake cycle and sleep structure, and the HPT axis is under the control of the central circadian pacemaker in the suprachiasmatic nucleus of the anterior hypothalamus [3]. Under normal sleeping conditions, plasma TSH levels begin to increase in the late afternoon or early evening before sleep, and reach a maximal concentration during the early part of the night [4]. Following the nocturnal peak, plasma TSH levels reduce during the rest of the sleep period, up to reach the low diurnal levels. Sleep has been demonstrated to exert an inhibitory influence on TSH production, as confirmed by the increase in hormonal secretion caused by sleep withdrawal [5]. In fact, TSH secretion, which is normally inhibited during sleep, continues to occur during nocturnal sleep deprivation determining higher morning plasma levels of the hormone compared to those found in subjects with a normal night sleep [1]. Additionally, recovery sleep, following withdrawal, suppresses TSH levels to larger extent than normal sleep [6].

Many individuals, particularly night shift workers, frequently undergo circadian misalignment by desynchronizing their sleep/wake cycle from the biological timing system. Thus, it is conceivable that shift-work intended by the International Labour Organization as a “a method of organization of working time in which workers succeed one another at the workplace” [7], and night-work defined by the European Union as “working at least 3 h of the daily shift or a certain proportion of the yearly working time in a period of 7 h defined by national law and including the time from midnight to 05:00” [8] may have a significant impact on thyroid function, in relation to circadian disruption, sleep deprivation, poor sleep efficiency, as well as sudden reversal of sleep/wake habits.

Shift-work, in particular night-work, in fact, has been recognized as stressors for the human body, as alterations of the sleep/wake cycle can affect human biological functions, physical–psychological conditions, quality of life, as well as working efficiency [9,10]. Overall, it has been demonstrated that shift-work is able to induce a huge spectrum of adverse health outcomes, ranging from sleep disturbances to the development of, e.g., cardiovascular, gastrointestinal, and neuropsychic diseases, and possible risk of cancers [11,12,13]. Less defined evidence is currently available on the relationship between circadian misalignment and adverse effects on metabolic and hormonal factors, particularly on the HPT axis. This seems an intriguing public and occupational health issue, considering the worldwide prevalence of shift-workers, as among one fifth of the workforce is involved into such kind of working schedule, and the 750 million people worldwide that the World Health Organization estimated to be affected by thyroid diseases, including cancer, hypothyroidism, hyperthyroidism and thyroiditis [14,15]. Therefore, the aim of the present study was to gain deep insight into the possible relationship between shift- and night-work and alterations in the function of the HPT axis, as well as the development of thyroid diseases. From an occupational risk assessment and management perspective, this may be useful to define early endocrine effects potentially induced by specific work schedules and particular conditions of hyper-susceptibility that should require peculiar preventive measures to protect the health of exposed workers.

## 2. Materials and Methods

The Preferred Reporting Items for Systematic Reviews and Meta-Analyses Statement (PRISMA) criteria were followed to perform a systematic literature search [16]. Studies addressing possible implications of “shift work” or “night work” on thyroid function or clinical outcomes, published until 20 January 2020, were identified by research on three principal scientific databases: PubMed, Scopus, and ISI Web of Science. The terms “shift work or night work” to assess the context of exposure and “thyroid” as the outcome of the investigation, combined with the Boolean operator “AND”, were employed for the research.

All the titles and abstracts retrieved through the computerized search were independently reviewed by three of the authors who selected papers suitable for the review purposes according to the inclusion criteria. These included all types of human peer-reviewed research articles (i.e., descriptive epidemiological-occupational surveys, medical reports, case series, cohort and case-control studies) published in English, and reporting possible implications of “shift work or night work” on various thyroid outcomes in workers. Exclusion criteria regarded reviews, case reports, conference papers, experimental studies on cellular and animal models, publications that did not focus on thyroid function of shift workers or those that, although exploring thyroid outcomes, did not provide information on shift schedules, as well as all the papers published in languages other than English.

The quality of the included studies was assessed separately by two investigators. Each eligible study was subjected to methodological critical appraisal through the Newcastle Ottawa Scale (NOS) adapted to perform a quality assessment of cross-sectional studies for systematic review [17,18,19]. The adapted NOS assesses the risk of bias in three domains: selection of participants (maximum 5 points); comparability of study groups (maximum two points); and outcome assessment (maximum 3 points). Quality rating of the studies was assessed as follows: very good (9, 10 points); good (7, 8 points); satisfactory (5, 6 points), unsatisfactory (0–4 points) (Table 1).

## 3. Results

**Figure 1:**
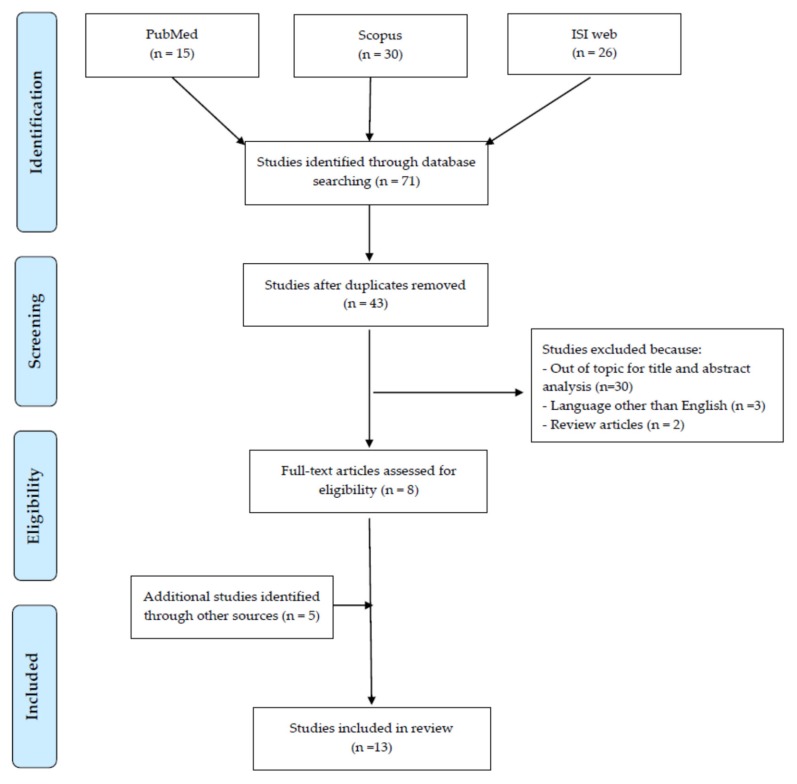
Flow diagram of literature search.

The preliminary search retrieved a total of 71 articles: 15, 30 and 26 in PubMed, Scopus, and Isi Web of Knowledge databases, respectively. From the total number of papers, 28 duplicates were removed. Among the remaining 43 articles, those (n. 35) that did not meet the inclusion criteria were excluded. Among those, 30 were considered out of the topic from the title and abstract analysis, 3 studies were excluded for the language other than English and 2 because were review articles. A total of 8 papers remained suitable for review. The full texts of all these articles were obtained and subjected to a critical evaluation. The citation pool of relevant publications was enlarged through the analysis of the reference list accompanying the selected articles identified with the previously detailed literature search. Another 5 eligible papers could be added. Overall, our search retrieved a total of 13 articles suitable for review (Figure 1). Most of the reviewed studies assessed the association between shift-work and night-work and thyroid hormonal changes in different occupational settings [20,21,22,23,24,25,26,27,28,29] (Table 1). Only a few publications focused on possible clinical adverse outcomes [26,30,31,32]. Some thyroid function alterations were demonstrated in relation to the shift-working organization and interesting issues could be pointed out as detailed in the following paragraphs.

### 3.1. Night Shift-Work and Thyroid Hormonal Changes

#### 3.1.1. Healthcare Sector

Several studies investigated changes in TSH levels in relation to night shift-work in the healthcare sector and found a positive correlation between such working schedule and increased hormonal concentrations [21,26,27]. Chang et al. [21] in a cohort of nurses enrolled in the largest psychiatric center in southern Taiwan, demonstrated that TSH levels were significantly higher in those who had sleep restriction for two consecutive night shifts compared to those who had been free of duty for at least 3 days before entering the study day. Comparably, in a retrospective study carried out on employees of a University hospital in Korea, Moon et al. [26] demonstrated that night shift workers had significantly 0.303 mIU/L higher TSH levels, over a 5 year study period (2011–2015), compared to non-night shift workers after adjusting for age and employment department. In line with these results, a study on Indian healthcare professionals, including doctors, nurses, technicians and support staff, demonstrated that mean TSH values were significantly higher in night shift (3.11 ± 1.81 mIU/L) than day-shift workers (2.04 ± 0.8 mIU/L) [27].

The effects of an irregular rotating working system, including night shifts, was also assessed in intensive care unit Greek nurses with respect to daily workers [23]. Thyroid stimulating hormone concentrations significantly decreased from the beginning to the end of a morning shift in rotating workers, while no significant effects were detected in the morning group. Conversely, T4 values significantly increased in rotating workers, but not in daily employees. When the overall mean changes in the hormonal levels were compared in the two groups, only T4 alterations resulted significantly different in rotating and daily workers. Concerning the possibility that irregular shifts may have effects on the circadian rhythm of serum hormone levels, and, consequently, on possible variables which are thought to modify an individual’s response to shift-work, such as personality, psychological health and social and domestic disruption, the authors found a significant positive correlation between TSH change in the rotating shift and the general health questionnaire score, indicating that greater changes in TSH are accompanied by poorer psychological health. Interestingly, when the TSH hormonal profile was assessed during a usual day sleep time for night- (7:00–15:00 h) and day-active workers (22:00–6:00 h), some differences could be pointed out [25]. In fact, TSH plateaued during night sleep in day-employees, while progressively declined during day sleep in night workers. Conversely, during working time, an ascending TSH trend was observed in night workers (22:00–6:00 h) in comparison to the lower levels seen in daytime ones (9:00–17:00 h). However, the mechanisms responsible for such changes as well as the physiological consequences of these hormonal profile modifications, still remain to be elucidated.

Hormonal changes have not been confirmed in a more recent study in healthcare workers, involved in rotating night shifts (principally nurses), and daily workers (primarily technicians) from an Italian teaching hospital, that showed comparable levels of TSH, FT3 and FT4 [32].

#### 3.1.2. Industrial Sector

Thyroid hormonal alterations were also investigated in shift-workers engaged in different industrial sectors, although with conflicting results. Among male workers employed in a rubber and ceramics Egyptian industry, who have been performing fixed morning-, afternoon- or night shifts for at least 2 years, those who were employed in night shifts had significantly higher levels of TSH than those who were employed in other schedules [28]. Night shift resulted as an independent predictor of TSH hormonal changes. These results are not in line with those obtained by a previous study carried out on female workers, employed in the packaging units of a pharmaceutical factory in Iran, that failed to demonstrate significant differences in TSH levels between night- and non-night shift workers [20].

### 3.2. Night on-Call-Work and Thyroid Hormonal Changes

When the effects of a night or 24-h on-call duty were investigated on the TSH levels, conflicting results were reported [22,24,25]. A significant increase in TSH levels was demonstrated by Harbeck et al. [22] in German internal medicine physicians the morning after a 24-h on-call-duty, compared to the TSH levels determined before a normal working day. Sleep deprivation with disturbed sleep patterns, natural biological rhythm desynchronization and stress conditions experienced in night-call-duties may stimulate nervous, immune and endocrine physiological responses, possibly leading to hormonal increase. Concerning the impact that such changes may have on physician neurocognitive functions and performance, no impairment was observed, while focused attention tended to be better after a 24-h shift, as a possible effect of long-term gating of attentional properties. This may support physician ability to adapt to the workload and stressful events during a 24-h on-call-shift, and TSH alterations may function as a first indicator of such an effect as other biochemical stress parameters, e.g., cortisol, epinephrine, norepinephrine, insulin and glucagon, did not show significant changes. An increasing trend in hormonal values was determined also in the study by Kuetting et al. [24] that observed significantly higher levels of TSH, FT3 and FT4 in German radiology residents following 24-h shift and work-related short-term partial sleep deprivation, compared to the values detected before the shift, while no significant changes were observed before and after a regular day of work and regular sleep.

When the effects of a night call-duty was assessed in Swedish physicians involved in different specialties, a significant 28% and 26% decrease in TSH levels were determined 1 day after the night call compared to those found in an ordinary day of work in anesthesiologists and pediatricians/ear-nose-throat surgeons, respectively [25]. In contrast to the anesthesiologists, the plasma levels of pediatricians/ear-nose-throat surgeons returned to baseline levels when measured 3 days after the night-call duty. Concerning FT4, a reduction of 3% was detected the third day after night call, compared with the plasma levels measured on the ordinary workday and 1 day after the night-call duty. However, the levels were within the normal range and the overall results did not imply serious metabolic changes with only minor differences between specialist groups.

### 3.3. Shift Work and Other Thyroid Effects

Limited evidence is currently available regarding thyroid diseases in shift- compared to non-shift workers [26,30,31,32]. Burdelack et al. [30] investigated the frequency of different diseases in a cohort of 725 nurses and midwives employed on rotating night shifts or in daily fixed shifts. They observed an overall prevalence of thyroid diseases of 21.2%, without significant percentage differences in rotating night shift workers (22.6%) and daily workers (20%). Interestingly, when the risk of thyroid disease was assessed according to the duration of night shift work, a twofold increased risk was detected in those workers employed for ≥15 years on night shifts in comparison to employees engaged in night shifts for a shorter, less than 15 years period of time. In the analysis of the relative risk of occurrence of thyroid diseases according to the frequency of night duties per month, no increase could be detected in workers with an average of more than 4 nights shifts per month with respect to those with less frequent night schedules. Unfortunately, this study failed to provide data on which diseases were considered in the analysis, therefore limiting the interpretation of the results. In the above-mentioned study by Moon et al. [26], the age-adjusted TSH levels ≥4.5 mIU/L among night shift healthcare workers were associated with a 1.4-fold higher risk of subclinical hypothyroidism compared to non-night shift workers. Comparably, a study carried out on 642 workers employed in a large teaching Italian hospital showed a significantly higher risk of subclinical autoimmune hypothyroidism in shift workers compared to the daytime workers, that remained significant also when possible confounding factors, such as age, gender, smoking habits, alcohol intake, familial history of autoimmune thyroid diseases, and exposure to radiations were included in the analysis [31]. Concerning anti-TPO antibodies prevalence, a significant difference was found between shift-workers, with a prevalence of 13.6%, compared to 8.6% of day-time workers. The prevalence of hypothyroidism unrelated to altered anti-TPO antibodies was significantly higher in shift workers (5.45%) than daytime workers (2.10%), while no differences could be detected for the prevalence of hyperthyroidism in absence of altered anti-TPO antibodies between the two investigated groups [31]. In a more recent study of the same research group [32], rotating night shift workers employed in the same hospital, showed a higher risk of thyroid nodules compared to diurnal workers. However, the lack of data on the characterization of such lesions and their possible malignancy, and information on potential co-exposures to other risk factors for thyroid diseases, such as ionizing radiation, do not allow to extrapolate definite conclusions.

## 4. Discussion

This review represents the first attempt to provide an overview on the possible alterations exerted by the circadian rhythm disruption induced by shift- and night shift-work in the function of the HPT axis and in the development of thyroid diseases. The determination of serum TSH concentrations has been employed as a specific and sensitive measure for the early detection of endocrine disorders. Different investigations performed on healthcare employees demonstrated that night shift-work could be related to higher levels of TSH serum levels in involved workers, although concentrations were included in a physiological range [21,22,24,26,27,28]. Interestingly, this effect was evident for any type of shift-working schedule considered, such as to have been involved in two consecutive night shifts before entry the study day [21], to have been performing more than 3 nights a week, for at least 1 year [27], as well as to have been engaged in night-, or 24-h on-call-duties [22,24].

Although the reason for such effects are not fully understood, it may be primarily conceived that night shift-induced changes in sleep schedule, timing, and quality may alter the body’s normal circadian rhythm and lead to an abnormal TSH circadian secretion. Changes in the TSH daily rhythmicity induced by sleep deprivation after night shifts may promote a hormonal increase [33,34]. It has also argued that night shift work can affect the immune system function, increasing the risk of autoimmune disease [35], as suggested by the results obtained by Moon et al. [26] and Magrini et al. [31] who determined a higher risk for subclinical autoimmune hypothyroidism in night shift workers compared to those not involved in such schedule. Additionally, several hormonal alterations, including those related to TSH secretion, may be dependent on the irregular eating habits and nocturnal eating frequent in night shift workers [36].

Although an increase in TSH serum concentrations was reported, the limited number of available studies and the noticeable heterogeneity in the enrolled populations, and shift-work scheduling, in terms of amount and type of shift- or night shift-work, does not allow to extrapolate definite conclusions, also, on possible underlining mechanisms of action. In this regard, it should be important to assess the role of personal features, including ethnicity, body mass index, calorie intake during work-shift, exercise status (including fitness level, sport activity performed before/after work or before going to bed), smoking and drinking habits, as well as therapies taken for comorbidities in possibly influencing TSH results. Particular attention in this regard should be focused at the worldwide aging of the workforce and the possible diverse age-dependent response that the endocrine system may have with respect to the circadian disruption induced by night shift-work. This seems important considering also the not definite evidence reporting an age-dependent increase in the median and upper reference limits of TSH [37,38,39,40], and an earlier shift of the beginning of the nocturnal rise of the serum TSH rhythm with aging [41]. Additionally, the impact of different work-related confounding factors, e.g., duration of employment, type of shift rotation, and sleep quality on hormonal response, should be also deeply evaluated. In contrast to classic rotating shift-work, in fact, physicians’ night call schedules are more irregular, with longer working hours (sometimes more than 16 h), reduced sleep and physiological and psychological stress, which may all function as specific factors influencing TSH alterations. On-call-duty was reported to induce disruption in the rhythm of TSH secretion even after a single night [22]. In this perspective, TSH may be the earliest and most sensitive parameter indicating even mild stress, whereas other biochemical stress indicators were less influenced by an on-call-shift, as also demonstrated by the lack of significant alterations in the concentrations of other circadian related hormones [22].

Overall, the different characteristics of the work-shifts may explain the not homogeneous results obtained on TSH levels. In fact, Rizza et al. [32] in healthcare professionals, and Attarchi et al. [20] in a pharmaceutical factory night shift workers failed to detect significant hormonal changes, while Korompeli et al. [23] and Marlberg et al. [25] determined a decreasing trend in TSH serum values in rotating shift nurses or in night-call-duty physicians, respectively. This latter result may be potentially related to a direct acute stress effect, or to the recovery sleep (following a period of sleep deprivation) characterized by a large fraction of deep sleep known to inhibit the hormone secretion [42]. It cannot be also excluded that the lack of TSH alterations could be explained by an “healthy worker” effect, meaning that those who could not face the workload had left the employment. Moreover, it is possible that participants had considerable experience on working on call and were probably confident in being able to handle emergency situations. These conditions may limit the stress experienced on such schedule organization, therefore preventing hormonal effects. However, deep details on work schedule, job tasks, duration of engagement in such kind of occupational organization, as well as population characteristics, in terms of physiological or pathological features, were not always provided by the authors, therefore preventing to clearly understand the relationship between thyroid functional changes and possible influencing occupational factors. These considerations may provide stimulus to future search aimed to deeply explore mechanisms responsible for specific hormonal changes also in relation to the characteristics of the work activities. In this regard, the possible effects of co-exposures to other risk factors on the thyroid function in the workplaces, e.g., chemical, biological and physical factors, should be considered. Although taking into consideration the different conditions of exposures and the remarkable inter-subject variability, the thyroid is very sensitive to the action of chemical disruptors [43]. Therefore, it cannot be ruled out that physicians, e.g., anesthesiologists and surgeons, employees in rubber and ceramic industry, as well as those involved in the packaging units of a pharmaceutical industry, may be exposed to chemicals, e.g., anesthetics, metals and plasticizers that may mediate hormonal alterations as reported in occupational, epidemiological and experimental settings [44,45,46,47,48,49].

Additionally, some other limitations of the studies should be carefully considered to have a suitable interpretation of the results. Several of the reviewed investigations focused on total female [20,21,26] or male [24] occupational populations. This limits the investigation of gender-based differences in sensitivity to night shift work-related hormonal changes in comparable workplace settings. Blood samples were obtained at different times, as workers have been evaluated during night or day shift depending on their duty schedules, therefore preventing achieving results representing specific changes in circadian rhythm well [26,28]. In some studies, differences between the beginning and the end of night shifts, or of the daytime shifts following the nights on duty, were assessed, therefore representing additional quite different outcomes for a correct interpretation of the endocrine effects. Moreover, all but one [21] of the reviewed studies did not collect blood samples on days off-duty, therefore preventing to achieve a complete picture of hormone profile on rest days. Moreover, in many cases, only TSH values were determined and this may limit a more comprehensive understanding of the effects that night shifts may have on thyroid functionality.

Concerning the role that such preclinical alterations may have in the possible development of thyroid diseases and the periods of time in which hormone alterations could be resolved, only one study confirmed that TSH levels, affected by short term sleep deprivation, returned quickly to control values following recovery sleep [25]. Interestingly, this hormonal trend was demonstrated only in particular groups of workers, e.g., pediatricians and ear-nose-throat surgeons compared to anesthesiologists, maybe in relation to their different job task features. In anesthesiologists, in fact, there may be an overlapping between the endocrine “insult” exerted by the circadian disruption induced by night shifts and strongly stressful events determined by taking care of life-threatening conditions for most of their working time, therefore providing an extra stress exposure. This may differently impact the thyroid function. The quick normalization of hormone values, on the one side, may suggest that such changes may most likely be without clinical significance. However, the findings of a higher prevalence of thyroid diseases [30], subclinical autoimmune hypothyroidism [26,31] and thyroid nodules [32] in night shift workers may suggest the need to deeply verify the long-term implications of early changes in hormonal pathways on endocrine diseases development. Additionally, it may be interesting to assess the relationship between night shifts, mental well-being, neurocognitive functions and performance of workers during different types of schedules, and thyroid hormonal changes. It should be verified whether determined hormone alterations may function as possible biomarkers of worker ability or “disability” to adapt to the workload and stressful events during night shifts or on-call-duties.

Although this review fills a gap in the literature, some limitations need to be considered when drawing conclusions from reported results. First of all, the cross-sectional nature of the studies inherently limits the definition of a suitable relationship between shift- or night-work and thyroid effects. The different conditions of exposure, as well as the variable outcomes investigated do not allow to easily integrate findings and extrapolate definite conclusions. According to the NOS checklist adapted for cross-sectional studies, about half of the reviewed papers were of satisfactory or good quality, while the others could be classified as unsatisfactory rating. Greater deficiencies regarded the representativeness of the exposed cohort, as enrolled subjects were generally selected groups of users and not sufficiently representative of the average target populations, as well as the inadequate degree of control for relevant confounding factors. Overall, although these aspects may question the reliability of the reviewed investigations, the relevance that this topic may have for the general 24-h workplace organization, as well as for the health of exposed workers, makes it important to explore the current, although still incomplete, knowledge on the topic with the aim to provide guidance for deeper future research.

## 5. Conclusions

These preliminary data on possible effects of shift-work, and particularly night-work, on the thyroid function of exposed workers need to be confirmed in future investigations designed to better define the relationship between shift schedules, in terms of, for example, night shifts per month, duration of employment in such organization, specific gender sensitivity, as well as possible confounding occupational factors and different endocrine outcomes, from early hormonal changes to confirmed clinical diseases. From an occupational health point of view, these findings may open innovative scenarios concerning workplace risk assessment and suitable measures to protect the health of night shift workers. Once confirmed, in fact, awareness concerning possible thyroid effects derived from the circadian disruption in night shift workers should require efforts for advanced approaches to risk assessment and management in workplace settings. Health surveillance programs should also include the evaluation of early thyroid alterations and specific attention to all subjects with possible hyper-susceptibility conditions. Considering also that about 10% of people worldwide are affected by thyroid diseases during their lifetime, with a definite need for therapy [50], the possible impact of shift-work on endocrine outcomes and the clinical history of the diseases appear extremely important to define fitness for work for affected workers. Overall this review provides stimulus to overcome knowledge gaps in the incompletely understood impact of shift-work organization on thyroid functionality of exposed workers. This will support the adoption and implementation of specific preventive strategies that may support a safer workplace organization and the timely, responsible realization of all the benefits of a 24-h economy.

## Figures and Tables

**Table 1:** Studies assessing the relationship between shift work and thyroid alterations.

Reference	Study Location	Study Design	Occupational Sector; Investigated Population	Outcome	Occupational Risk Factors	Additional Information	Results	Quality Rating According to NOS
Chang et al. [21]	Southern Taiwan	Cross sectional	*Healthcare sector* Female nurses involved in 2 continuous night shifts (*n* = 20; mean age ± SD: 26.0 ± 2.0 years); Off duty nurses who had been free of duty for at least 3 days before entering the study (*n* = 23; mean age 26.1 ± 1.9 years)	Hormonal changes	Night shift-work	*Working schedule* All subjects had worked daytime shifts, evening-shifts, or had been free of duty for at least 3 days before entering the study day. All subjects had been working in the hospital for at least one year. *Biological sampling* Blood samples were collected and tested for TSH at the end of a night shift or on the off-duty day. The test was repeated 4 times every 2 h starting at 9:20 am.	Significant increase in TSH level in night shift workers (1.3 ± 0.7; 1.5 ± 0.7, 1.9 ± 0.9, 2.0 ± 0.7 mIU/L at morning time 1 and 2, afternoon time 1 and 2, respectively) compared to off-duty workers (1.0 ± 06, 1.0 ± 06, 1.0 ± 06, 1.1 ± 0.6 mIU/L, at morning time 1, and 2, afternoon time 1 and 2, respectively).	Unsatisfactory
Moon et al. [26]	Incheon, Korea	Cross sectional	*Healthcare sector* Total female adult workers (*n* = 967; age range: ≤29–≥ 50 years); night shift workers (*n* = 546); non-night shift workers (*n* = 421).	Hormonal changes	Night shift-work	*Working schedule* Night shift workers: ≥4 night shifts per month. *Departmental status* Nurses (*n* = 654): general ward, outpatient unit, emergency room, operating room, and/or intensive care unit; workers in other departments (*n* = 313): support workers or administrative workers.	No significant differences in TSH annual mean levels between night shift workers (3.27 mIU/L) and non-night shift workers (2.98 mIU/L). Significant increase in TSH levels of night shift workers (0.303 mIU/L) compared to non-night shift workers at the GEE analysis adjusted for age and department. Risk of subclinical hypothyroidism (TSH levels of 4.5 ≥ mIU/L): 1.399-fold higher risk in night shift workers compared to their non-night shift workers.	Satisfactory
Sathyanarayana et al. [27]	Bangalore, India	Cross sectional	*Healthcare sector* Doctors, nurses, technicians and support staff (n. 40). Day-shift workers (*n* = 20; mean age 34.25 ± 8.16 years); Night shift workers (*n* = 20; mean age 27.50 ± 5.14 years)	Hormonal changes	Night shift-work	*Working schedule* Day-shift workers: schedule timings between 8:00 and 17:00 Night shift workers: schedule timings between 20:00 and 8:00 Night shift workers had ≥3 night shifts per week and monthly night shift working hours of 60–90 h. Participants had a minimum of 1 year working experience in these schedules. *Biological sampling* Blood sample to determine the serum TSH was collected after 10–12 h of fasting between 7:30 and 8:30 am.	Significant increase in TSH levels in night shift workers (3.11 ± 1.81 mIU/L) compared to day-shift workers (2.04 ± 0.8 mIU/L).	Unsatisfactory
Korompeli et al. [23]	Athens, Greece	Cross sectional	*Healthcare sector* Total intensive care unit nurses (*n* = 32; mean age: 36.7 ± 1.2 years); Rotating shift group (*n* = 25; 13 females, 12 males); Exclusively morning-shift group (*n* = 7, 2 males, 5 females).	Hormonal changes	Irregular rotating shifts, including night shifts	*Working schedule* Rotating shift group (morning: 7 am–3 pm; evening: 3–11 pm; night shifts 11 pm–7 am); Exclusively morning shift group (7 am–3 pm); All nurses had been working for more than 3 years in an intensive care unit. *Biological sampling* Blood samples were collected from each participant at the beginning and at the end of the shift.	Significant decrease in TSH concentrations from the beginning and the end of the shift for rotating workers (2.34 ± 0.32 vs 1.78 ± 0.27 mIU/L). No significant differences in morning group (1.67 ± 0.44 vs. 1.36 ± 0.30 mIU/L) Significant reduction of T3 values from the beginning and the end of the shift for morning workers (1.17 ± 0.04 vs. 1.05 ± 0.05 ng/mL), non-significant differences in rotating workers (1.06 ± 0.04 vs 1.01 ± 0.04 ng/mL). Significant increase in the mean T4 concentrations from the beginning and the end of the shift in rotating workers (7.10 ± 0.25 vs. 7.35 ± 0.22 μg/dL, respectively); non-significant differences in morning workers (7.40 ± 0.25 vs. 7.26 ± 0.26 μg/dL, respectively).	Unsatisfactory
Weibel and Brandenberger [29]	Strasbourg, France	Cross sectional	Night workers (*n* = 11 males; age range: 24–25 years); Day-active workers (*n* = 8; age range 23–32 years)	Hormonal changes	Night shift-work	*Working schedule* Workers had been working on night shift (4–5 consecutive night shifts per week) for at least 2 years; No naps were allowed during the night work. *Biological sampling* Workers were studied during their usual 24-h sleep-wake cycle following day sleep from 7 am–15 pm in night shift workers and the nocturnal sleep 23 pm–7 am sleep period in daytime workers.	*Hormonal profile during usual sleep time*: TSH concentrations plateaued during night sleep (23:00–6:00 h) among day-active subjects and declined progressively during day sleep (07:00–15:00 h) among night shift workers, with a significant different slope between the 2 groups (Spearman’s R: −0.07 ± 0.17 vs. −0.57 ± 0.09, respectively). *Hormonal profiles during usual work time:* TSH were low among day-active subjects (09:00–17:00 h), but increased among night workers (22:00–6:00 h), with significant different slope between the 2 groups (Spearman’s R: −0.59 ± 0.11 vs. 0.74 ± 0.05, respectively)	Unsatisfactory
Harbeck et al. [22]	Luebeck, Germany	Prospective crossover study	*Healthcare sector* Physicians working in department of internal medicine (*n* = 20; 11 females, 9 males; median age: 32 years)	Hormonal changes	Twenty-four hours on-call-occupational shift	*Working schedule* Each physician completed a 24-h control period including a regular 8 h non-on-call-shift and a 24-h on-call-shift 2–4 weeks later. Working experience ranged from 1–2 to >than 4 years. *Biological sampling* Biochemical parameters were assessed at 8:00 am prior to a normal working day and, 2–4 weeks later, at 8:00 am of the morning following a 24-h on-call shift in internal medicine.	Significant increase in TSH levels were detected the morning after the 24-h on-call-duty (2.9 ± 1.7 mIU/L) compared to the TSH levels determined in the same group before a normal working day (2.0 ± 0.8 mIU/L).	Unsatisfactory
Kuetting et al. [24]	Bonn, Germany	Prospective cohort study	*Healthcare sector* Radiology residents (*n* = 20; 19 males, 1 female; mean age: 31.6 ± 2.1 years); Controls from the same group (*n* = 10)	Hormonal changes	Twenty-four hours on-call-occupational shift	*Working schedule* Twenty-four-hour shift with an average of 3 h of sleep. *Biological sampling* Examinations were performed before and after the 24-h on-call-duty and before and after a normal day of 9 h of work in controls (at least 7 days from the 24-h on-call-duties).	Significant increase in TSH (2.44 ± 0.9 mIU/L), FT3 (3.4 ± 0.4 pg/mL) and FT4 (1.0 ± 0.1 ng/dL) values following 24-h on-call-duty, in comparison to concentrations detected before the on-call-shift (TSH: 1.54 ± 0.5 mIU/L; FT3: 3.1 ± 0.20 pg/mL; FT4: 0.92 ± 0.1 ng/dL). No significant changes in TSH (1.53 ± 0.3 vs. 1.516 ± 0.4 mIU/L), FT3 (3.03 ± 0.2 vs. 3.12 ± 0.19 pg/mL) and FT4 (0.97 ± 0.01 vs. 0.95 ± 0.05 ng/dL) before and after a regular day of work (9 h) and a regular sleep (at least 6 h).	Unsatisfactory
Malmberg et al. [25]	Lund, Sweden	Cross sectional	*Healthcare sector* Anesthesiologists (*n* = 19; females: 39%; median age 42 years; median years’ experience on night call: 9 years); Pediatricians and ear-nose-throat surgeons (*n* = 18; females: 59%; median age 39 years; median years’ experience on night call: 10 years)	Hormonal changes	Night-call duty	*Working schedule* Night-call duty started at approximately 16:00 h and lasted for about 16 h. Anesthesiologists and pediatricians: separate night call weeks (2–3 nights) every fourth to sixth week. Ear-nose-throat surgeons: single nights on call every second or third week. *Biological sampling* Blood samples were collected on an ordinary workday, 1 and 3 days after work on a night call.	Significant reduction in mean TSH levels (26%–28%) one day after night-call duty (anesthesiologists: 1.37–95% CI 1.04–1.70; pediatricians and ENT surgeons: 1.43–95% CI 1.06–1.79 mIU/L) compared to ordinary days of work (anesthesiologists: 1.83-CI 95% 1.51–2.14; pediatricians and ENT surgeons: 1.92-CI95% 1.56–2.29 mIU/L). Three day after the night-call duty: anesthesiologists: 1.52-CI 95% 1.19–1.86 mIU/L; pediatricians and ear-nose-throat surgeons: 2.03-CI95% 1.67–2.40 mIU/L.	Satisfactory
Shaker et al. [28]	Cairo, Egypt	Cross sectional	*Industrial sector* Total male investigated workers (*n* = 99; mean age: 46.8 ± 10.0 years); Fixed morning-shift (*n* = 36); Fixed afternoon-shift (*n* = 19); Fixed night- shift (*n* = 44) (median age range 25–60)	Hormonal changes	Shift-work	*Working schedule* The factory was engaged in the manufactures of railway sleepers, rubber, pottery, and cement bricks. Workers were employed in fixed morning, afternoon or night shifts for at least 2 years. *Biological sampling* Blood samples were collected in the morning at the workplace.	Significant increase in median TSH values in night shift workers (2.8 mIU/L; IQR 2.2–3.6 mIU/L) than in morning (0.7 mIU/L; IQR 0.5–0.9 mIU/L) and afternoon-shift workers (1.4 mIU/L; IQR 1.2–1.7 mIU/L).	Satisfactory
Attarchi et al. [20]	Tehran, Iran	Cross sectional	*Industrial sector* Total female workers in packaging units of a pharmaceutical industry (*n* = 406; mean age 31.3 years); Shift workers (*n* = 113 years); Non-shift-workers (*n* = 293).	Hormonal changes	Shift-work	*Working schedule* No data are available on shift-work organization; All women working in the packaging units of the factory had at least one year of work experience. *Biological sampling* Blood samples for hormone analysis were collected on the third day of menstruation.	No significant differences in mean TSH levels were detected between shift workers (2.98 ± 0.70 mIU/L) and non-shift workers (2.89 ± 0.98 mIU/L).	Satisfactory
Burdelak et al. [30]	Lodz, Poland	Cross sectional	Female nurses and midwives (*n* = 725; age range: 40–60 years); Rotating night shift workers (*n* = 354; median age: 48 years); Day workers (*n* = 371; median age: 50 years)	Thyroid diseases	Shift-work and night shift-work	*Working schedule* Women working rotating on night shifts had been working at night for an average of 25.4 years; Women working days only, but who had had a history of night shift-work, had, on average, worked for 12 years on night shifts. Out of daily workers, the majority (83%) had changed to day work more than 5 years before their recruitment into the study.	No significant difference in the prevalence of thyroid diseases between night shift and daily workers. Thyroid disease prevalence in the total investigated population: 21.2%; in shift workers: 22.6%; in day workers. 20%. The risk of thyroid disease was higher in ≥15 year night shift-workers compared to <15 year night shift workers (OR = 1.97; 95% CI: 1.21–3.20)	Satisfactory
Magrini et al. [31]	Rome, Italy	Cross sectional	Workers in a large teaching hospital (*n* = 642); Shift workers (n. 220; 81 females, 129 males, radiation exposed *n* = 81; mean age: 32.67 ± 7.29 years); Day-time workers (*n* = 422; 222 females, 200 males; radiation exposed 95; mean age: 40.17 ± 11.32)	Thyroid diseases	Shift work	*Working schedule* Day time workers: shift from 6 AM to 6 PM; Shift workers: involved in rotating shifts, including night shifts.	Significant increase in subclinical autoimmune hypothyroidism prevalence in shift workers (7.7%) and in day-time workers (3.8%). OR for shift workers: 2.26 (CI 95% 1.02–4.98) Significant higher prevalence of abnormal anti-TPO antibodies level in shift workers (13.6%) than in day-time workers (8.6%). No significant differences in hypothyroidism and hyperthyroidism unrelated to altered anti-TPO antibodies in shift- and day-time workers (5.45% vs 2.10%; 2.84% vs 1.36%, for hypo and hyperthyroidism, respectively).	Good
Rizza et al. [32]	Rome, Italy	Retrospective cohort study	*Healthcare sector* Workers employed in a teaching hospital (*n* = 299); Rotating night shift workers (*n* = 160; 51 males; mean age 38.7 ± 9.4 years); Day-workers (*n* = 139; 39 males; mean age 36.5 ± 7.9 years).	Thyroid diseases	Shift-work	*Working schedule* Rotating night shift workers: shift schedule of four up to seven 12 h nights per month, followed by 2 days off; mean length of employment in night shifts: 9 ± 4 years; Day workers: had never worked night shifts.	No significant differences in TSH, FT3, FT4 and anti-TPO levels between rotating night and day- workers. Night shift work was statistically related to thyroid nodules (odds ratio: 1.78; 95%CI 1.09–3.23). Nodule size and the percentage of multiple nodules were similar in both groups.	Satisfactory

AM—ante meridiem; anti-TPO—anti-thyroid peroxidase; CI—confidence interval; FT3—free-triiodothyronine; FT4—free-thyroxine, GEE—generalized estimating equation; IQR—interquartile range; NOS—Newcastle Ottawa Scale; PM—post meridiem; SD—standard deviation; TSH—thyroid stimulating hormone.
